# Combined Heart and Kidney Transplantation: Clinical Experience in 100 Consecutive Patients

**DOI:** 10.1161/JAHA.118.010570

**Published:** 2019-02-12

**Authors:** Morcos Atef Awad, Lawrence S. C. Czer, Dominic Emerson, Stanley Jordan, Michele A. De Robertis, James Mirocha, Evan Kransdorf, David H. Chang, Jignesh Patel, Michelle Kittleson, Danny Ramzy, Joshua S. Chung, J. Louis Cohen, Fardad Esmailian, Alfredo Trento, Jon A. Kobashigawa

**Affiliations:** ^1^ Division of Cardiology Cedars‐Sinai Smidt Heart Institute the Multiorgan Transplant Program Cedars‐Sinai Medical Center Los Angeles CA; ^2^ Division of Cardiothoracic Surgery Department of Surgery Cedars‐Sinai Smidt Heart Institute the Multiorgan Transplant Program Cedars‐Sinai Medical Center Los Angeles CA; ^3^ Division of Pediatric Nephrology the Multiorgan Transplant Program Cedars‐Sinai Medical Center Los Angeles CA; ^4^ Section of Biostatistics Cedars‐Sinai Medical Center Los Angeles CA; ^5^ Department of Surgery Cedars‐Sinai Medical Center Los Angeles CA

**Keywords:** heart failure, heart transplantation, hemodialysis, kidney transplantation, mortality, renal disease, Heart Failure, Cardiomyopathy, Transplantation, Mortality/Survival

## Abstract

**Background:**

Combined heart and kidney transplantation (HKTx) is performed in patients with severe heart failure and advanced renal insufficiency. We analyzed the long‐term survival after HKTx, the influence of age and dialysis status, the rates of cardiac rejection, and the influence of sensitization.

**Methods and Results:**

From June 1992 to December 2016, we performed 100 HKTx procedures. We compared older (≥60 years, n=53) with younger (<60 years, n=47) recipients, and recipients on preoperative dialysis (n=49) and not on dialysis (n=51). We analyzed actuarial freedom from any cardiac rejection, acute cellular rejection, and antibody‐mediated rejection, and survival rates by sensitized status with panel‐reactive antibody levels <10%, 10% to 50%, and >50%, and compared these survival rates with those from the United Network for Organ Sharing database. There was no difference in 15‐year survival between the 2 age groups (35±12.4% and 49±17.3%, ≥60 versus <60 years; *P*=0.45). There was no difference in 15‐year survival between the dialysis and nondialysis groups (44±13.4% and 37±15.2%, *P*=0.95). Actuarial freedom from any cardiac rejection (acute cellular rejection>0 or antibody‐mediated rejection>0) was 92±2.8% and 84±3.8%, acute cellular rejection (≥2R/3A) 98±1.5% and 94±2.5%, and antibody‐mediated rejection (≥1) 96±2.1% and 93±2.6% at 30 days and 1 year after HKTx. There was no difference in the 5‐year survival among recipients by sensitization status with panel‐reactive antibody levels <10%, 10% to 50%, and >50% (82±5.9%, 83±10.8%, and 92±8.0%; *P*=0.55). There was no difference in 15‐year survival after HKTx between the United Network for Organ Sharing database and our center (38±3.2% and 40±10.1%, respectively; *P*=0.45).

**Conclusions:**

HKTx is safe to perform in patients 60 years and older or younger than 60 years and with or without dialysis dependence, with excellent outcomes. The degree of panel‐reactive antibody sensitization did not appear to affect survival after HKTx.

**Clinical Perspective**

**What Is New?**

- Renal failure after heart transplantation is associated with increased mortality, but combined heart and kidney transplantation remains controversial because of the shortage of donor organs.
- In 100 consecutive patients with heart and kidney transplantation at a single institution, 15‐year survival rates were similar in older and younger recipients (≥60 and <60 years) and those with and without pretransplant dialysis, with a survival rate in the entire cohort similar to the United Network for Organ Sharing registry over a comparable time period.
- Sensitization (panel‐reactive antibody levels <10%, 10% to 50%, and >50%) did not appear to influence survival in these patients, all of whom received induction therapy.

**What Are the Clinical Implications?**

- Heart and kidney transplantation is safe and feasible, with excellent outcomes in older and younger patients, with or without dialysis, and in sensitized patients.
- Heart and kidney transplantation should continue to be offered to patients with advanced heart failure and renal insufficiency.

## Introduction

The number of patients undergoing heart and kidney transplantation (HKTx) has increased in recent years,^1^ as well as the number of waitlisted patients for HKTx.^2^ While on the waitlist, the 3‐month mortality rate of HKTx‐listed patients was observed to be 21% in dialysis‐dependent patients and 7% in nondialysis‐dependent patients with renal insufficiency.^2^ Analysis of the United Network for Organ Sharing (UNOS) registry showed similar mortality rates between heart transplantation (HTx)‐ and HKTx‐listed patients, but the 5‐year survival rates of HKTx recipients were higher than the survival rates of HTx recipients with renal insufficiency regardless of the pretransplant dialysis dependence status. Another analysis of the UNOS registry showed higher survival rates in HKTx recipients than in HTx recipients requiring pretransplant dialysis,^3^ and further analysis of the UNOS database provided an association between pre‐HTx estimated glomerular filtration rate (GFR) and end‐stage renal disease, kidney transplantation, and mortality for up to 10 years after HTx.^4^, ^5^ A pre‐HTx estimated GFR <60 mL/min per m^2^ was associated with increased mortality after HTx.^4^ Thus, lower GFR portended higher renal risks and mortality after isolated HTx.^2^, ^3^, ^4^, ^5^, ^6^ These findings suggest that concomitant heart failure and renal insufficiency warrants consideration for HKTx when the estimated GFR is <60 mL/min per m^2^, without necessitating dialysis dependence. In patients with pre‐HTx renal dysfunction with abnormal GFR, one also needs to consider the increased risk of end‐stage renal disease following HTx because of the cumulative effects of calcineurin inhibitor nephrotoxicity^6^ and the attendant increase in post‐HTx mortality if end‐stage renal disease develops.^1^, ^4^

We have previously shown that combined HKTx is a safe and effective approach in selected patients with combined heart failure and advanced renal insufficiency.^7^, ^8^, ^9^, ^10^ There is experimental evidence to suggest that the greater mass of transplanted tissue after HKTx may reduce rejection rates and promote graft tolerance.^8^, ^10^ Sensitized patients have worse outcomes after isolated heart or kidney transplantation,^11^, ^12^ but the outcome after combined HKTx is unknown.

The aim of this study was to analyze the outcomes of HKTx recipients at our center stratified by age older and age younger than 60 years and by presence or absence of pretransplant dialysis. We also aimed to analyze the frequency of rejection in our HKTx recipients, and the outcomes of HKTx recipients based on their panel‐reactive antibody (PRA) sensitization level. Finally, we compared the survival rates of our HKTx cohort with the HKTx and HTx cohorts of the UNOS registry over a similar time period.

## Methods

From June 1992 to December 2016, a total of 100 HKTx procedures were performed at our institution. From February 1992 to December 2014, UNOS registered 42 426 HTx and 777 HKTx recipients. We classified our 100 patients based on 2 criteria: age older and age younger than 60 years and whether the patient received dialysis before HKTx. We compared the demographics, preoperative characteristics, and survival outcomes. We also analyzed the rates of acute cellular rejection (ACR) and antibody‐mediated rejection (AMR) on the endomyocardial biopsies of the study population at our center. In addition, we compared our 100 HKTx recipients with the HTx and HKTx recipients in the UNOS registry. UNOS registry data were obtained from a Standard Transplant Analysis and Research (STAR) file.

Orthotopic heart transplantation was performed at our institution using the usual surgical technique, either with a biatrial or bicaval anastomosis.^13^, ^14^, ^15^, ^16^, ^17^ Patients were hemodynamically stabilized in the operating room or intensive care unit before kidney transplantation.^8^, ^9^, ^10^ The details of anti‐human leukocyte antigen antibody detection using cell‐based or solid‐phase assays, PRA, desensitization methods, immunosuppression regimen, infection prophylaxis, and follow‐up echocardiography, coronary angiography, endomyocardial biopsy, and detection of AMR and ACR at our institution were previously described.^7^, ^8^, ^9^, ^10^, ^11^, ^12^, ^18^, ^19^, ^20^, ^21^, ^22^, ^23^ All patients received induction therapy consisting of intravenous muromonab‐CD3 (5 mg) or horse anti‐thymocyte globulin (15 mg/kg) for 7 days (before January 2000) or rabbit anti‐thymocyte globulin (1.5 mg/kg) for 5 to 7 days (after January 2000).^8^, ^9^, ^10^, ^19^, ^20^, ^21^, ^22^

Before July 2007, we used a prospective complement‐dependent cytotoxicity crossmatch between donor and recipients in sensitized patients (PRA >10%); however, we started routinely using virtual crossmatch after July 2007.^18^ We showed that virtual crossmatch could shorten the wait time on the HTx waitlist without increasing rejection or mortality rates postoperatively. We continued to use a prospective complement‐dependent cytotoxicity crossmatch in more sensitized (PRA >50%) and in highly sensitized (PRA >70%) patients to ensure a negative cytotoxic crossmatch before transplant. Flow cytometric crossmatches were also performed, both prospectively and retrospectively.

We collected data about our HKTx recipients in a deidentified fashion. Function of the recipient heart before transplant was analyzed using left ventricular ejection fraction, left ventricular end‐diastolic diameter, cardiac output, and cardiac index (CI), and whether cardiac intervention, in the form of inotropic support, intra‐aortic balloon pump (IABP), or mechanical circulatory support (MCS) (either ventricular assist device [VAD] or total artificial heart [TAH], was required for patient hemodynamic stability. Renal function was analyzed using creatinine levels and the dialysis status of patients. Analyzed cardiac risk factors included history of coronary artery disease, peripheral vascular disease, hypertension, hyperlipidemia, diabetes mellitus, and smoking.

The grades of ACR and AMR were identified based on the pathological findings of endomyocardial biopsy after transplantation, as previously described.^8^, ^19^, ^20^, ^21^, ^22^ The degree of ACR followed the International Society for Heart and Lung Transplantation (ISHLT) grading system^24^, ^25^: grade 0 for no rejection, grade 1R (1A, 1B, and 2) for mild rejection, grade 2R (3A) for moderate rejection, and grade 3R (≥3B) for severe rejection. The degree of AMR followed the ISHLT grading system^26^: grade 0 for no rejection, grade 1 for mild rejection, grade 2 for moderate rejection, and grade 3 for severe rejection. Combined AMR and ACR rejection was classified as shown in Table 1. Sensitization status was analyzed based on PRA levels <10%, 11% to 50%, and >50%.

**Table 1:** Rejection Classification in Heart and Kidney Transplant Recipients

Rejection Classification	ACR and AMR Classification	No. of Patients
No rejection	ACR 0 and AMR 0	82
Mild cellular	ACR 1R and AMR 0	5
Mild antibody‐mediated	ACR 0 and AMR 1	3
Mild mixed	ACR 1R and AMR 1	0
Moderate cellular	ACR 2R and AMR 0/1	4
Moderate antibody‐mediated	ACR 0/1R and AMR 2	4
Moderate mixed	ACR 2R and AMR 2	0
Severe cellular	ACR 3R and AMR 0/1	2
Severe antibody‐mediated	ACR 0/1 and AMR 3	0
Severe mixed	ACR 2R/3R and AMR 3; or ACR 3R and AMR 2/3	0

ACR indicates acute cellular rejection; AMR, antibody‐mediated rejection.

Data from the UNOS registry were received in a deidentified fashion regarding HTx and HKTx recipients from February 1992 to December 2014. The study was approved by the institutional review board, and the requirement for informed consent was waived. As previously noted, the UNOS registry data were obtained from a STAR file provided by UNOS. The data from our institution are included in the STAR file. The data, analytic methods, and study materials will not otherwise be available to other researchers for purposes of reproducing the results or replicating the procedure.

Continuous variables are presented as mean±SD, while categorical or integer variables are presented as number and percentage. To compare values, *t* test was used for normally distributed numerical variables, while Wilcoxon rank sum test was used for nonnormally distributed numerical variables. Fisher exact test was used for comparison of categorical variables. Rates of survival and freedom from rejection were estimated by Kaplan–Meier method and compared using log‐rank test. A value of 0.05 was used for significance throughout. SPSS version 18.0 (SPSS Inc) and SAS version 9.4 (SAS Institute) were used for statistical analysis. In the analysis of age and survival, proportional hazards assumption was assessed by Supremum Test (in SAS version 9.4), which was negative, with *P*=0.43.

## Results

**Figure 1:**
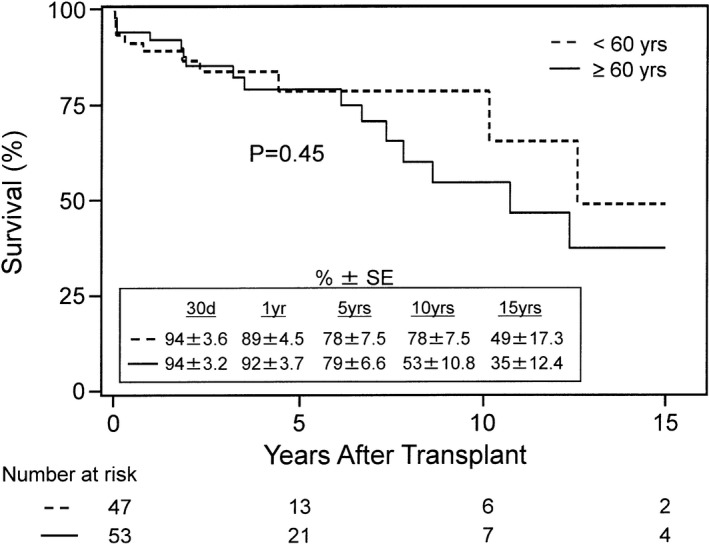
Kaplan–Meier survival rates after heart and kidney transplantation in recipients younger (<) and older than (≥) 60 years. Survival rates were similar between the groups up to 15 years after transplant (*P*=0.45). SE indicates standard error.

Table 2 shows the demographics and preoperative characteristics of HKTx recipients stratified into 2 groups by age: the older group (≥60 years, n=53) and the younger group (<60 years, n=47). Patients in the older group had lower creatinine levels (3.03±1.46 versus 5.00±3.57 mg/dL, *P*=0.0009) when compared with those in the younger group, and there were also fewer patients in the older group on dialysis (35.8% versus 63.8%, *P*=0.009). However, there was no difference in left ventricular ejection fraction, left ventricular end‐diastolic diameter, cardiac output, and CI between both groups (*P*=0.41, 0.33, 0.94, and 0.72, respectively). There was also no difference in the New York Heart Association classification or etiologies of cardiomyopathy among patients in the 2 groups (*P*=0.88 and 0.16, respectively). In addition, both groups had a similar frequency of patients requiring inotropic support, IABP, and MCS (*P*=0.84, 0.24, and 0.16, respectively); however, there was a higher frequency of patients with diabetes mellitus in the older group (45.3% versus 23.4%, *P*=0.035). Figure 1 shows the Kaplan–Meier survival curve for patients in the older and younger groups after HKTx. There was no significant difference in the overall survival rates between both groups at 15 years (35±12.4% and 49±17.3%, *P*=0.45), especially during the initial 7 years following HKTx.

**Table 2:** Demographics and Preoperative Variables of Heart and Kidney Transplant Recipients Stratified by Age

Variable	Overall (N=100)	Age ≥60 y (n=53)	Age <60 y (n=47)	*P* Value
Age at treatment, y	57.3±10.8	65.1±3.4	48.4±9.3	NA
Men	83 (83.0)	45 (84.9)	38 (80.9)	0.61
Height, cm	173.7±8.5	173.3±8.6	174.2±8.4	0.59
Weight, kg	77.2±17.9	75.7±15.6	79.0±20.2	0.36
BMI, kg/m^2^	25.48±5.20	25.07±4.21	24.69±5.41	0.41
UNOS status 2	25 (25.0)	12 (22.6)	13 (27.7)	0.65
NYHA class				
II	4 (4.0)	2 (3.8)	2 (4.3)	0.88
III	32 (32.0)	16 (30.2)	16 (34.0)	
IV	64 (64.0)	35 (66.0)	29 (61.7)	
Cardiomyopathy				
Ischemic	61 (61.0)	37 (69.8)	24 (51.1)	0.16
Idiopathic	30 (30.0)	12 (22.6)	18 (38.3)	
Other	9 (9.0)	4 (7.6)	5 (10.6)	
LVEF, %	29.7±17.0 (n**=**98)	28.4±16.6	31.2±17.5 (n**=**45)	0.41
LVEDD, mm	59.4±13.4 (n**=**93)	60.7±12.6 (n**=**49)	58.0±14.3 (n**=**44)	0.33
CO, L/min	4.77±1.52 (n**=**94)	4.76±1.30 (n**=**49)	4.79±1.75 (n**=**45)	0.94
CI, L/min per m^2^	2.50±0.70 (n**=**93)	2.53±0.65 (n**=**49)	2.47±0.76 (n**=**44)	0.72
MCS	24 (24.0)	16 (30.2)	8 (17.0)	0.16
Inotropic support	41 (41.0)	21 (39.6)	20 (42.6)	0.84
IABP	12 (12.0)	8 (15.1)	4 (8.5)	0.24
Prior sternotomy	66 (66.0)	32 (60.4)	34 (72.3)	0.29
Creatinine, mg/dL	3.95±2.82 (n**=**99)	3.03±1.46	5.00±3.57 (n**=**46)	0.0009
Dialysis	49 (49.0)	19 (35.8)	30 (63.8)	0.009
CAD	65 (65.0)	37 (69.8)	28 (59.6)	0.3
PVD	11/83 (13.3)	7/42 (16.7)	6/41 (9.8)	0.52
Hypertension	72 (72.0)	39 (73.6)	33 (70.2)	0.82
Hyperlipidemia	52/99 (52.5)	32 (60.4)	20/46 (43.5)	0.109
Diabetes mellitus	35 (35.0)	24 (45.3)	11 (23.4)	0.035
Smoking	38/99 (38.4)	25 (47.2)	13 (28.3)	0.064
Obesity	21 (21.0)	8 (15.1)	13 (27.7)	0.145
Alcohol	15 (15.0)	11 (20.8)	4 (8.5)	0.101

Continuous numerical variables are represented as mean±SD, and integer or categorical values as number (percentage). BMI indicates body mass index; CAD, coronary artery disease; CI, cardiac index; CO, cardiac output; IABP, intra‐aortic balloon pump; LVEDD, left ventricular end‐diastolic diameter; LVEF, left ventricular ejection fraction; MCS, mechanical circulatory support; NA, not available; NYHA, New York Heart Association; PVD, peripheral vascular disease; UNOS, United Network for Organ Sharing.

**Figure 2:**
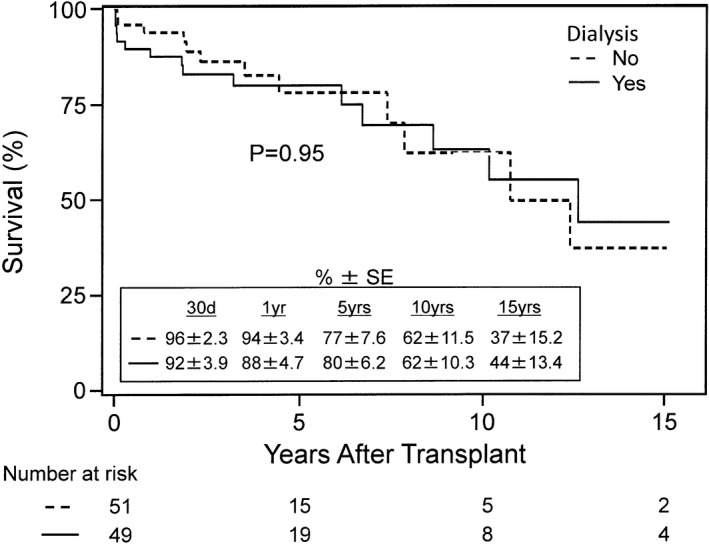
Kaplan–Meier survival rates after heart and kidney transplantation in recipients with and without pretransplant dialysis. Survival rates were similar between the groups up to 15 years after transplant (*P*=0.95). SE indicates standard error.

Table 3 shows the demographics and preoperative characteristics of HKTx recipients stratified into 2 groups by pre‐HKTx dialysis: patients on dialysis (n=49) and patients not on dialysis (n=51). On average, there were younger patients in the dialysis group (*P*=0.004). Patients on dialysis had higher pre‐HKTx creatinine levels compared with those not on dialysis (5.32±3.47 versus 2.60±0.65, *P*<0.0001). Importantly, there was no difference in left ventricular ejection fraction, left ventricular end‐diastolic diameter, cardiac output, and CI between the 2 groups (*P*>0.99, 0.81, 0.90, and 0.97, respectively). There was also no difference in the New York Heart Association classification and etiologies of cardiomyopathy among patients in both groups (*P*=0.23 and 0.13, respectively). In addition, both groups had a similar frequency of patients requiring inotropic support (*P*=0.69); however, there was a trend toward a higher frequency of patients in the dialysis group who were on IABP or MCS (*P*=0.069 and 0.06, respectively). Figure 2 shows the Kaplan–Meier survival curve for patients on dialysis and not on dialysis before HKTx. There was no difference in the overall 15‐year survival rates between the 2 groups (44±13.4% and 37±15.2%, *P*=0.95).

**Table 3:** Demographics and Preoperative Variables of Heart and Kidney Transplant Recipients Stratified by Pretransplant Dialysis Status

Variable	Overall (N=100)	Dialysis (n=49)	No Dialysis (n=51)	*P* Value
Age at treatment, y	57.3±10.8	54.2±10.5	60.2±10.3	0.004
Age ≥60 y	53 (53.0)	19 (38.8)	34 (66.7)	0.009
Men	83 (83.0)	40 (81.6)	43 (84.3)	0.79
Height, cm	173.7±8.5	172.6±7.4	174.8±9.4	0.19
Weight, kg	77.2±17.9	75.0±18.3	79.4±17.4	0.22
BMI, kg/m^2^	25.48±5.20	25.05±5.47	25.89±4.94	0.42
UNOS status 2	25 (25.0)	13 (26.5)	12 (23.5)	0.82
NYHA class				
II	4 (4.0)	3 (6.1)	1 (2.0)	0.23
III	32 (32.0)	12 (24.5)	20 (39.2)	
IV	64 (64.0)	34 (69.4)	30 (58.8)	
Cardiomyopathy				
Ischemic	61 (61.0)	29 (59.2)	32 (62.7)	0.13
Idiopathic	30 (30.0)	18 (36.7)	12 (23.5)	
Other	9 (9.0)	2 (4.1)	7 (13.7)	
LVEF, %	29.7±17.0 (n**=**98)	29.7±17.0 (n**=**47)	29.7±17.3	>0.99
LVEDD, mm	59.4±13.4 (n**=**93)	59.1±13.1 (n**=**46)	59.8±13.9 (n**=**47)	0.81
CO, L/min	4.77±1.52 (n**=**94)	4.75±1.61 (n**=**47)	4.79±1.44 (n**=**47)	0.90
CI, L/min per m^2^	2.50±0.70 (n=93)	2.50±0.77 (n=46)	2.50±0.63 (n=47)	0.97
MCS	24 (24.0)	16 (32.7)	8 (15.7)	0.06
Inotropic support	41 (41.0)	19 (38.8)	22 (43.1)	0.69
IABP	12 (12.0)	9 (18.4)	3 (5.9)	0.069
Prior sternotomy	66 (66.0)	35 (71.4)	31 (60.8)	0.3
Creatinine, mg/dL	3.95±2.82 (n=99)	5.32±3.47	2.60±0.65 (n=50)	<0.0001
CAD	65 (65.0)	30 (61.2)	35 (68.6)	0.53
PVD	11/83 (13.3)	6/40 (15.0)	5/43 (11.6)	0.75
Hypertension	72 (72.0)	35 (71.4)	37 (72.5)	>0.99
Hyperlipidemia	52/99 (52.5)	23 (46.9)	29/50 (58.0)	0.32
Diabetes mellitus	35 (35.0)	15 (30.6)	20 (39.2)	0.41
Smoking	38/99 (38.4)	17 (34.7)	21/50 (42.0)	0.54
Obesity	21 (21.0)	10 (20.4)	11 (21.6)	>0.99
Alcohol	15 (15.0)	5 (10.2)	10 (19.6)	0.26

Continuous numeric variables are represented as mean±SD and integer or categorical values as number (percentage). BMI indicates body mass index; CAD, coronary artery disease; CI, cardiac index; CO, cardiac output; IABP, intra‐aortic balloon pump; LVEDD, left ventricular end‐diastolic diameter; LVEF, left ventricular ejection fraction; MCS, mechanical circulatory support; NYHA, New York Heart Association; PVD, peripheral vascular disease; UNOS, United Network for Organ Sharing.

**Figure 3:**
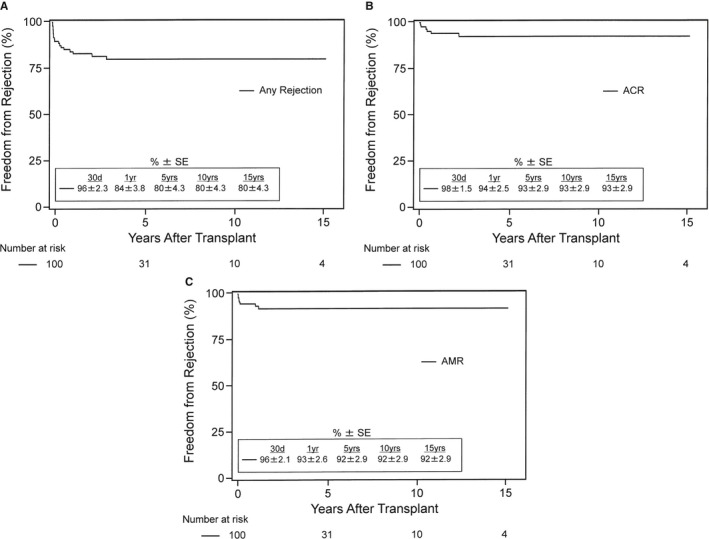
Kaplan–Meier actuarial freedom from rejection after heart and kidney transplantation. **A**, free from any cardiac rejection (grade of acute cellular rejection [ACR] >0 or antibody‐mediated rejection [AMR] >0), (**B**) free from significant or treated cellular rejection (ACR ≥2R/3A), (**C**) free from AMR (AMR ≥1). SE indicates standard error.

Of the 100 HKTx recipients in the study, 18 patients had allograft rejection: 11 with ACR >0 and 7 with AMR >0. Of the 11 patients with ACR, 4 had grade 1R (1A), 1 had grade 1R (1B), 4 had grade 2R (3A), 1 had grade 3R (3B), and 1 had grade 3R (3B or 4) rejection. The frequency of patients with ACR or AMR is shown in Table 1. Figure 3 shows the Kaplan–Meier curves of the freedom from any rejection (Figure 3A), ACR ≥2R (Figure 3B), and AMR≥1 (Figure 3C). Actuarial freedom from any rejection was 92±2.8% and 84±3.8% at 30 days and 1 year, respectively, and 80±4.3% at 5, 10, and 15 years following HKTx. Actuarial freedom from ACR ≥2R was 98±1.5% and 94±2.5% at 30 days and 1 year, respectively, and 93±2.9% at 5, 10, and 15 years following HKTx. Actuarial freedom from AMR ≥1 was 96±2.1% and 93±2.6% at 30 days and 1 year, respectively, and 92±2.9% at 5, 10, and 15 years following HKTx.

**Figure 4:**
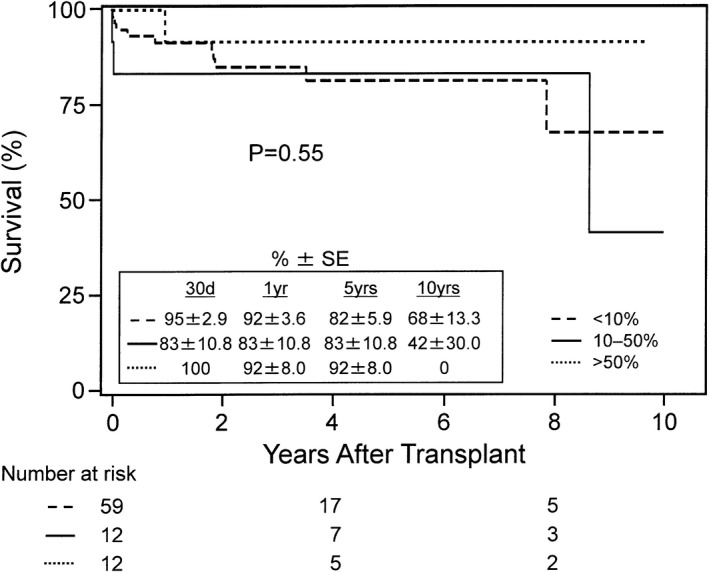
Kaplan–Meier survival rates after heart and kidney transplantation in recipients with panel‐reactive antibody sensitizations <10%, 10% to 50%, and >50%. Survival rates were similar between the groups up to 5 years after transplant (*P*=0.55). SE indicates standard error.

Of the 100 HKTx recipients in the study, PRA data on sensitization were obtained in 83 patients: 59 patients with PRA <10%, 12 patients with 10% to 50%, and 12 patients with >50% sensitization. Figure 4 shows the Kaplan–Meier survival curves for HKTx recipients stratified into the aforementioned 3 groups. There was no difference in the overall 5‐year survival rates among the 3 groups (*P*=0.55). The survival rates of patients with PRA <10% were 95±2.9% at 30 days and 82±5.9% at 5 years after HKTx, while the survival rates of patients with PRA 10% to 50% were 83±10.8% at 30 days and 5 years after HKTx. For patients with PRA >50%, the survival rates stayed at 92±8.0% from 1 to 5 years after HKTx.

**Figure 5:**
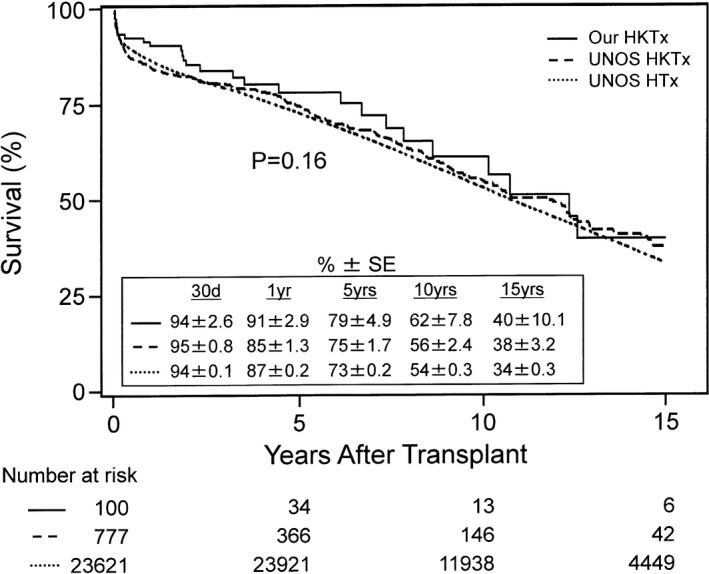
Kaplan–Meier survival rates comparing the United Network for Organ Sharing (UNOS) heart transplantation (HTx) experience, the UNOS heart and kidney transplantation (HKTx) experience, and our HKTx experience. Our data are from June 1992 to December 2016. The UNOS data are from February 1992 to December 2014. Survival rates were similar among all groups up to 15 years after transplant (*P*=0.16). Survival rates were also similar between UNOS HKTx and our own HKTx recipients up to 15 years after transplant (*P*=0.45 when separately analyzed). SE indicates standard error.

Figure 5 shows the Kaplan–Meier survival curves comparing our HKTx experience with the UNOS database for HKTx and HTx. Of note, the 30‐day (early) survival rates were comparable among all groups at 94±2.6%, 95±0.8%, and 94±0.1%, respectively. The overall 15‐year survival rates of our HKTx experience (40±10.1%), the UNOS HKTx experience (38±3.2%), and the UNOS HTx experience (34±0.3%) were similar among all of the groups (*P*=0.16, Figure 5) and when comparing both HKTx experiences with each other (*P*=0.45, Figure 5).

## Discussion

In a recent study of the UNOS registry, only 171 of 637 (26.8%) HKTx recipients were 60 years or older.^2^ In the current study of 100 HKTx recipients, 53 patients (53%) were older than 60 years, with a mean age of 65.1±3.4 years. HKTx recipients younger than and those older than 60 years had a similar frequency of preoperative cardiac risk factors, with the exception of a higher frequency of diabetes mellitus in the older group. It is worth noting that the mean age of HKTx recipients did not differ (*P*=0.91) from the initial 17 years (n=30, 57.4±12.1 years) to the subsequent 7 years (n=53, 57.6±10.5 years) of our HKTx experience.^10^ In the current study, we showed no difference in survival rates, for up to 15 years postoperatively, between recipients younger than and those older than 60 years, even though the younger group of patients had similar cardiac function and worse renal function compared with the older group of patients. Of note, the mean CI of the 93 of 100 HKTx recipients was 2.50±0.70 L/min per m^2^ compared with the mean CI of 2.48±0.77 L/min per m^2^ in the 637 HKTx recipients in the UNOS registry.^2^ Based on the current analysis, age younger or older than 60 years does not appear to have an effect on mortality for up to 15 years after HKTx, given the careful selection of patients considered for combined organ transplantation, a topic discussed in previous publications.^7^, ^9^

The current study also showed no difference in survival rates, for up to 15 years postoperatively, between HKTx recipients with and without dialysis preoperatively, even though recipients requiring dialysis were younger than but had similar cardiac function as recipients not requiring dialysis. The prioritization of allocation of kidneys to heart transplant recipients is a matter of contention, given the large number of patients with end‐stage renal disease awaiting cadaveric renal allografts. It seems that the degree of preoperative renal dysfunction, based on elevated creatinine levels and dialysis requirement, does not affect survival following HKTx. The opposite, however, seems to be true when comparing HKTx with HTx alone. A recent analysis of the UNOS registry showed that in dialysis‐dependent patients or in patients with nondialysis‐dependent renal insufficiency, the survival rates of HKTx recipients (73% and 80%, respectively) were improved compared with the survival rates of HTx recipients (51% and 69%, respectively) at 5 years postoperatively.^2^ This study also showed that in a multivariable analysis, preoperative dialysis dependence or creatinine clearance <50 mL/min did not predict postoperative outcomes in HKTx recipients. In addition, dialysis requirements preoperatively did not appear to affect delayed renal graft function (defined as the need for dialysis within the first week) postoperatively in HKTx recipients.^27^ Taken together, these findings imply that the combined HKTx procedure overcomes the survival disadvantage of preoperative renal dysfunction (estimated GFR <60 mL/min per 1.74 m^2^) with HTx alone, and concurrent allocation of dual organs in these recipients appears appropriate. This has important implications for organ allocation.

In the current study, cardiac function before HKTx, based on left ventricular ejection fraction, cardiac output, and CI, was comparable between patients with or without dialysis, given that some of those patients were on IABP or MCS (VAD or TAH). A total of 24 patients (24%) and 12 patients (12%) in this population were on MCS and IABP, respectively, before HKTx in comparison with the frequency of HKTx recipients reported in a recent UNOS registry analysis who required preoperative VAD support (17.3%) or mechanical (IABP, ventilator, ECMO, or VAD) support (23.9%).^2^ In this current report, there was a trend toward an increased frequency of patients on IABP (18.4% versus 5.9%, *P*=0.069) or MCS (32.7 versus 15.7, *P*=0.06) in the dialysis group. At least 2 of those patients developed dialysis dependence after TAH implantation, both of whom had a stage 3 chronic kidney disease before implantation of TAH. Dialysis was initiated urgently secondary to cardiogenic shock in 1 patient and secondary to hemodynamic decline while on inotropic support in the other patient.^28^ Both patients subsequently underwent HKTx with good postoperative allograft recovery.

Given the significant proportion of HKTx recipients who have been on some form of mechanical or VAD support before transplant (41%),^2^ it is important to examine the impact of this therapy on outcomes after HKTx, including possible effects on graft function and rejection. An analysis of the UNOS registry showed that requiring preoperative mechanical life support (IABP, VAD, ECMO, or ventilator) predicted post‐HKTx mortality.^2^ However, a more recent analysis of the UNOS registry showed similar survival rates in HKTx recipients with and without preoperative continuous flow LVAD at 1 year (77% versus 82%) and 3 years (75% versus 77%) after HKTx, respectively.^29^ MCS therapy was associated with delayed renal graft function (defined as the need for dialysis within the first week) after HKTx.^27^ In a recent study, we did not find an association between preoperative MCS and delayed renal graft function, morbidity, or mortality after HKTx.^30^ In the HTx patient population, a recent study found an association, on multivariable analysis, between preoperative VAD support and the need for acute postoperative renal replacement therapy.^5^

Interestingly, a recent study suggested that preoperative serum immunoglobulin G polyreactive natural antibody levels were significantly elevated in HTx recipients who were on VAD support compared with their counterparts.^31^ This elevation in serum immunoglobulin G natural antibodies was associated with postoperative primary graft dysfunction. Additionally, non‐human leukocyte antigen antibodies have been associated with MCS therapy and the appearance of de novo anti‐human leukocyte antigen antibodies after HTx.^32^, ^33^, ^34^, ^35^, ^36^ It remains to be determined whether the same holds true after combined HKTx, and whether this has a detrimental or protective effect on the combined transplant procedure. Also, it is not known whether the type of MCS (eg, TAH versus VAD) has different effects on post‐transplant outcomes after combined HKTx.^28^, ^37^

In the current analysis of 100 HKTx recipients, we report a 30‐day freedom from any rejection (ACR >0 or AMR >0) of 92±2.8%, with 98±1.5% freedom from ACR ≥2R and 96±2.1% freedom from AMR ≥1. We also report a 5‐year freedom from any rejection of 80±4.3%, with 93±2.9% freedom from ACR ≥2R and 92±2.9% freedom from AMR ≥1. In the current analysis, 18 of 100 HKTx recipients experienced rejection: 7 had AMR and 11 had ACR. Of those with ACR, 5 patients had grade 1R rejection and 6 patients had ACR ≥2R (previous ISHLT grades 3A, 3B, and 4). This is the largest single‐center study to report and differentiate rates of cardiac ACR and AMR after HKTx. A previous analysis of the UNOS registry involving 263 HKTx recipients showed an acute cardiac rejection rate of 14.5% and an acute renal rejection rate of 6.5% at 1 year after transplant.^3^ All HKTx recipients in the current study received induction therapy,^8^, ^9^, ^10^ which may account for the low rates of ACR and AMR in this study. A possible survival advantage to the use of induction therapy with ATG (anti‐thymocyte globulin)^19^, ^20^, ^38^ in HKTx recipients has been recently shown in the UNOS registry, especially in sensitized patients maintained on tacrolimus, mycophenolate mofetil, and prednisone at the time of hospital discharge.^39^

In the current study, pre‐HKTx screening for anti‐human leukocyte antigen antibodies showed 59 of 83 patients (71%) with PRA <10%, 12 of 83 patients (14.5%) with PRA 10% to 50%, and 12 of 83 patients (14.5%) with PRA >50%. Thus, a significant proportion of patients undergoing HKTx (29%) in the current study were sensitized (PRA ≥10%). By comparison, in a recent UNOS registry study of 263 HKTx recipients, 49.8% of recipients had a peak PRA <10%, 4.6% of recipients had peak PRA 10% to 30%, and 4.2% of recipients had a peak PRA >30%.^3^

According to ISHLT registry data published in 2017, the frequency of sensitization (PRA >10%) in the HTx recipient population has increased significantly in recent years.^1^, ^40^, ^41^, ^42^ Of 8160 HTx recipients in a recent analysis of the UNOS registry, increasing PRA levels were associated with rejection in the first year after HTx, with rejection rates at 32% to 38% in recipients with PRA ≤25% and 45.5% in recipients with PRA >25%. The survival rates of HTx recipients with PRA ≤25% were higher than those with PRA >25%, with 5‐year survival being 73.9% compared with 65.3%, respectively, and increasing PRA (and thus greater sensitization) was identified as a predictor of mortality after HTx.^43^ Other studies have shown a similar association between sensitization and post‐transplant mortality among HTx recipients in a multivariate analysis^44^ and in the ISHLT registry data published in 2017.^1^, ^40^, ^41^, ^42^

In our study, we found no difference in survival among HKTx recipients with PRA <10%, 10% to 50%, and >50%. This could be related to the initiation of virtual crossmatching, which was started after July 2007.^18^ We found in sensitized HTx recipients that virtual crossmatching was associated with a shorter waiting time on the transplant list with no additional negative effect on ACR, AMR, or mortality after HTx.^18^ Additionally, we continued to use prospective complement‐dependent cytotoxicity crossmatching in highly sensitized HTx and HKTx recipients, since it may be a better predictor of early rejection after transplant than the virtual crossmatch, thus allowing judicious donor selection. Lastly, the routine use of induction therapy in the current study may be ameliorating the higher risk of rejection from sensitization, as implied in the recent UNOS registry analysis of HKTx recipients.^39^ More studies are needed to better identify factors associated with improved survival after HKTx.

## Study Limitations

Limitations to this study include the inherent selection bias in the multidisciplinary approach of evaluating potential candidates for HKTx. Another limitation is that we did not stratify post‐HKTx rejection by organ rejection: heart or kidney or both. In addition, we did not include an analysis of rejection rates from the UNOS registry of HTx and HKTx recipients. Finally, when comparing our institution's outcomes with the UNOS registry outcomes, there was a difference in the time of data collection between both cohorts: our experience represents data from June 1992 to December 2016, whereas the UNOS registry experience represents data from February 1992 to December 2014. Finally, a portion of the results were from a single institution, which may limit the generalizability of the results.

## Conclusions

We report an analysis of 100 HKTx recipients, stratified by age 60 years and older and age younger than 60 years, and by presence or absence of pretransplant dialysis. There was no difference in overall 15‐year survival rates between older and younger recipients, given that older recipients had lower pretransplant creatinine levels and frequency of dialysis. There was no difference in overall 15‐year survival rates between recipients with and without pretransplant dialysis, given that patients on dialysis were younger on average. Actuarial freedom from any rejection, ACR ≥2R, and AMR ≥1 was 80±4.3%, 93±2.9%, and 92±2.9%, respectively, for up to 15 years after transplantation. There was no difference in the overall 5‐year survival rates between recipients with pretransplant PRA levels <10%, 10% to 50%, and >50%. In addition, there was no difference in overall 15‐year survival rates between our HKTx experience and the UNOS HKTx and HTx experiences. Performing HKTx on a select group of patients older than or younger than 60 years and with or without dialysis‐dependent renal insufficiency is feasible and safe with excellent outcomes that are comparable to the national average. According to this analysis, pretransplant PRA sensitization levels do not appear to affect survival rates after HKTx; however, we used virtual crossmatching in combination with preoperative complement‐dependent cytotoxicity crossmatching in sensitized recipients, all of whom received induction therapy. Large‐scale trials or analyses are required to further exploit the benefit of different types of crossmatching and the benefit of induction therapy in HKTx.

## Disclosures

Jignesh Patel reports research funding from Sanofi Genzyme. David Chang reports research funding from Amgen and Mesoblast, and equity interest in Abbott, Abbv, and Repligen. The remaining authors have no disclosures to report.
